# Epicardial–Endocardial Reentry in Ischemic Cardiomyopathy

**DOI:** 10.19102/icrm.2021.120402

**Published:** 2021-04-15

**Authors:** Yasuhito Kotake, Chrishan J. Nalliah, Timothy Campbell, Ivana Trivic, Neil Ross, Richard G. Bennett, Samual Turnbull, Saurabh Kumar

**Affiliations:** ^1^Department of Cardiology, Westmead Hospital, Sydney, Australia; ^2^Westmead Applied Research Centre, University of Sydney, New South Wales, Australia

**Keywords:** Ablation, ischemic cardiomyopathy, ventricular tachycardia

## Abstract

In ischemic cardiomyopathy, endocardial reentry has traditionally been the mechanistic paradigm for understanding ventricular tachycardia (VT). However, recognition is growing that epicardial myocardium is a critical component for VT substrate, even in patients with ischemic cardiomyopathy. In this report, we present a novel case of a three-dimensional VT reentry involving epicardial components and an endocardial exit.

## Case presentation

A 53-year-old man with a history of ischemic heart disease presented with conscious monomorphic ventricular tachycardia (VT), having had multiple shocks delivered by a dual-chamber implantable cardioverter-defibrillator (ICD). He had experienced anterior ST-elevation myocardial infarction three years previously, with successful percutaneous coronary intervention of the mid–left anterior descending artery. Following the percutaneous coronary intervention, his left ventricular (LV) ejection fraction was estimated at 36%, warranting ICD implantation. Antiarrhythmic drug therapy at initial presentation was a β-blocker, with amiodarone 200 mg twice daily additionally commenced prior to discharge.

**Figure 1:**
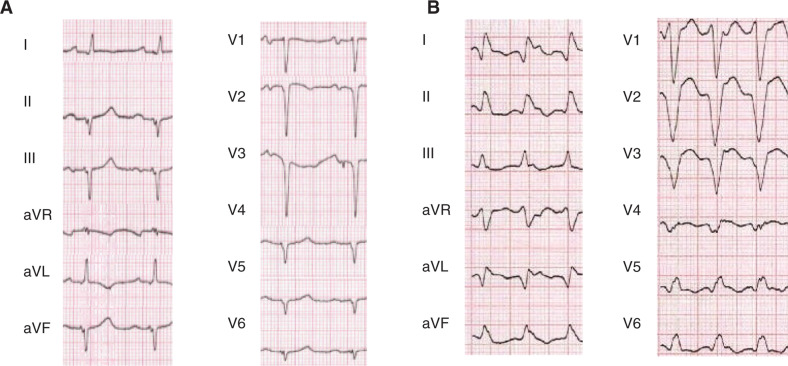
Twelve-lead ECG. **A:** Sinus rhythm. **B:** Ventricular tachycardia.

During the subsequent 18 months, he presented with recurrent, hemodynamically tolerated sustained monomorphic VT that was treated by the ICD (both antitachycardia pacing and shock therapy). A sinus rhythm electrocardiogram (ECG) showed anterior Q-waves consistent with a previous anterior transmural infarction **(Figure 1A)**. His clinical VT morphology as follows remained quite similar at each presentation: left bundle branch block morphology, inferior axis, precordial transition at V_4_/V_5_, and a biphasic (sr) pattern in the left lateral leads with a tachycardia cycle length of 420 to 460 ms. These morphologic features in combination with the prior history of anterior infarction suggested an exit from the LV anteroseptum/right ventricular (RV) outflow tract **(Figure 1B)**.

**Figure 2:**
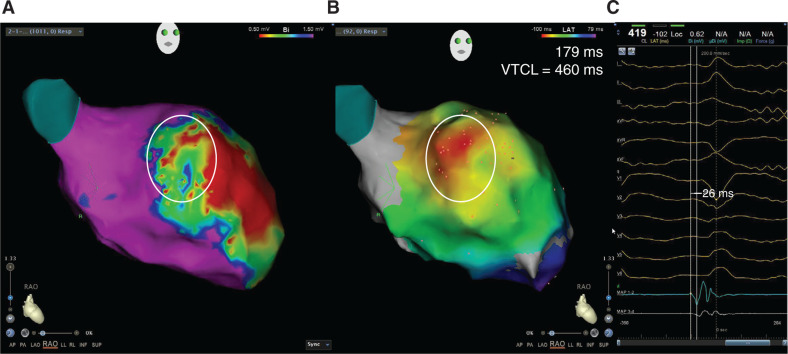
First ablation session: voltage, activation mapping, and earliest electrogram. **A:** LV endocardial voltage mapping. **B:** LV endocardial activation mapping. **C:** Earliest electrogram.

During the 18-month period, endocardial ablation was attempted several times; however, VT with a similar morphology recurred after each attempt. During the first procedure, endocardial LV mapping observed a large region of low bipolar voltage area over the LV anterior wall containing fractionated potentials **(Figure 2A)**. VT activation mapping localized the exit to the scar border zone of the LV anteroseptum **(Figure 2B)**. The earliest site of LV endocardium during VT activation mapping was −26 ms pre-QRS onset, although only 179 ms of the 460-ms tachycardia cycle length was sampled in the LV **(Figure 2C)**. Extensive endocardial ablation was performed over the anteroseptum region, targeting abnormal potentials and regions of early activation.

**Figure 3:**
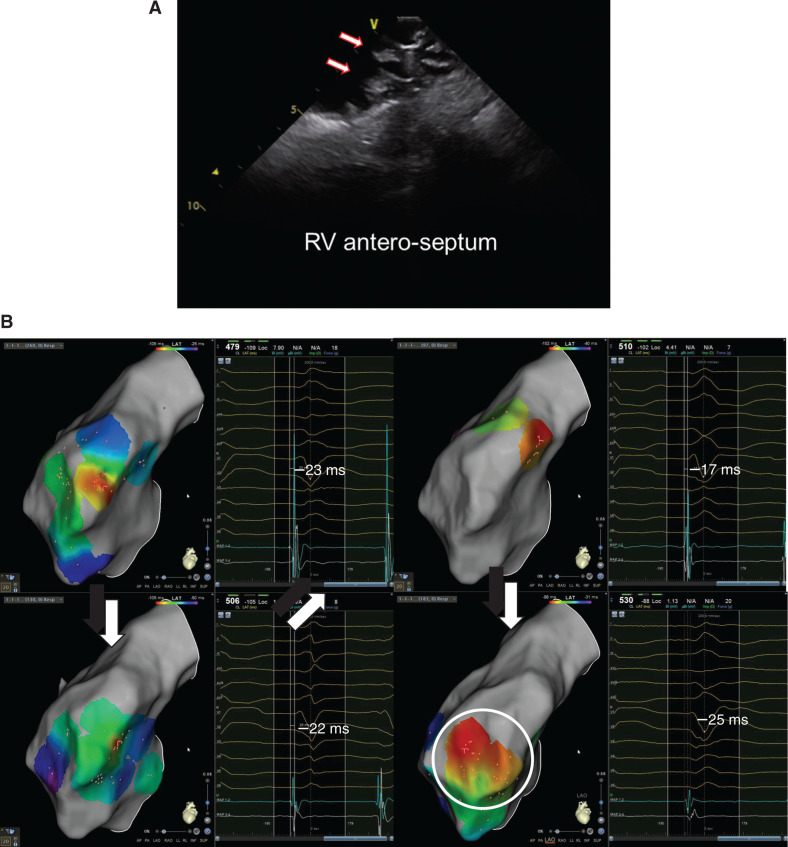
Second ablation session: varying exit sites during the procedure and trabeculated anteroseptal RV. **A:** Trabeculated anteroseptal RV. **B:** Varying exit sites during the procedure.

The second procedure predominantly used pacemapping due to the difficulty inherent in maintaining the tachycardia for activation mapping. Once more, extensive ablation was performed at the distal aspect of the scar border. The most recent attempt at ablation involved mapping of the RV, LV, and coronary cusps. The RV was noted to be highly trabeculated, with the earliest activation occurring at the RV free wall **(Figure 3A)**. Notably, there were no low-voltage zones in the RV. Radiofrequency ablation at this region initially suppressed the VT, yet it remained inducible with varying subtle morphology variations **(Figure 3B)** and recurred spontaneously shortly thereafter. Activation mapping observed a broad region of early activation of the right endocardial surface. Overall, the earliest region of endocardial activation was at the RV free wall 25 ms ahead of the surface ECG QRS. Ablation at this site suppressed the tachycardia and the procedure was stopped due to its long duration; however, VT recurred one week later.

**Figure 4:**
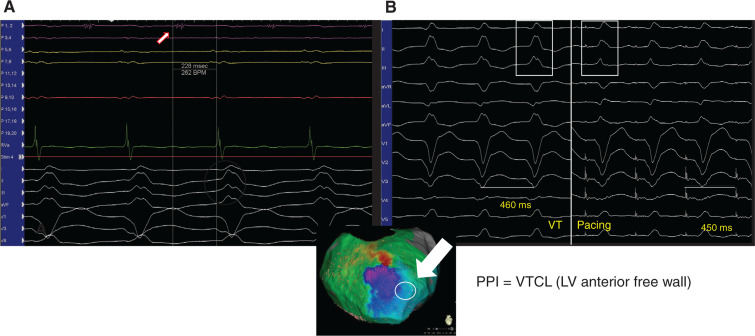
Third ablation session: mid-diastolic signals and manifest fusion (epicardial approach). **A:** Mid-diastolic signals. **B:** Entrainment with manifest fusion. Note, the PPI equals the VTCL (LV anterior free wall).

**Figure 5:**
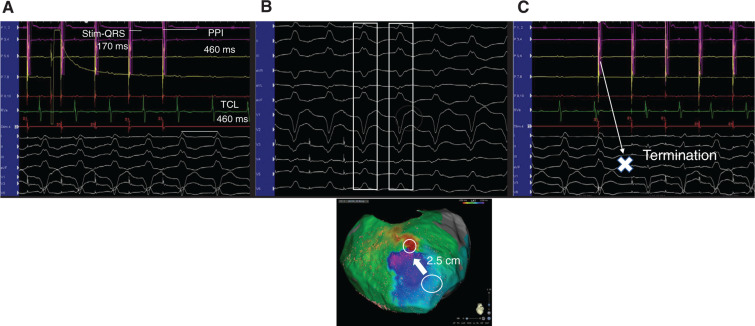
Third ablation session: concealed fusion and termination without global capture. **A and B:** Entrainment with concealed fusion. **C:** Termination without global capture. Note, the PPI equals the VTCL and Stim-QRS interval is 37% (170/460 ms) of the VTCL (2.5 cm superior to the previous point/LV anterior free wall).

**Figure 6:**
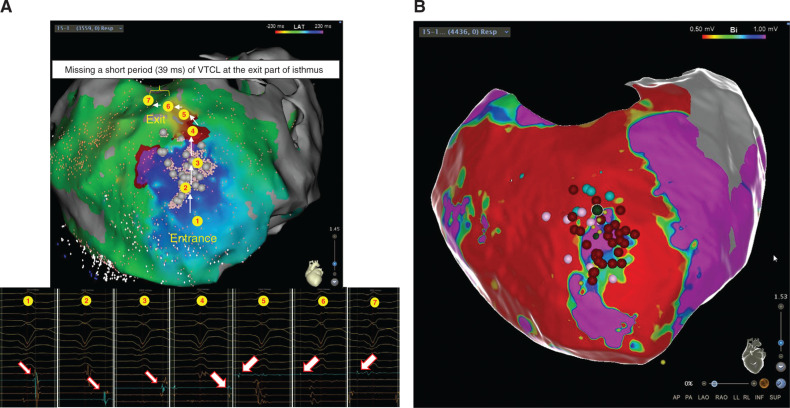
Third ablation session: epicardial activation mapping and voltage mapping with ablation. **A:** Epicardial activation mapping. **B:** Epicardial voltage mapping with ablation.

At this point, a third ablation was performed. VT activation mapping of the RV endocardium revealed early activation at the prior ablation site. However, entrainment maneuvers confirmed that this site was distant from the putative reentrant circuit (ie, the postpacing interval minus the tachycardia cycle length was 283 ms). Epicardial mapping observed broad regions of multiple, long, continuous, and mid-diastolic potentials over the intraventricular septum **(Figure 4A)**. Manifest fusion was observed during entrainment from the distal anterior LV free wall with a postpacing interval consistent with a pacing site within the outer loop of the circuit **(Figure 4B)**. Approximately 2.5 cm superior to this point, we were able to identify the site of interest, which was concealed fusion with a postpacing interval placing the pacing site within the isthmus of the circuit (ie, the postpacing interval equaled the tachycardia cycle length) **(Figure 5A and 5B)**. The stimulation–QRS interval measured approximately 37% (170/460 ms) of the VT cycle length, indicating that the pacing site was within the central portion of the VT isthmus,^1^ and was located at the scar border zone on the anterior LV free wall. Localization within the VT isthmus was further confirmed by VT termination without global capture during additional entrainment attempts **(Figure 5C)**, suggesting a location within the critical isthmus.^2^ A review of the activation map showed a period of cycle length discontinuity, while the review of biventricular endo- and epicardial activation maps showed that 39 ms of the tachycardia cycle length as the tachycardia exited the epicardium into the endocardium was missing, suggesting an intramural mechanism **(Figure 6A)**. High-density epicardial voltage mapping revealed a channel of surviving tissue extending into the scar. Ablation was performed at this site, homogenizing the surviving myocardium that extended into the region of scar **(Figure 6B)**. Entrainment confirming presence within the circuit at two points located at least 2 cm apart established a macro-reentrant mechanism. Activation mapping illustrated a slowed activation at the putative isthmus at the scar border zone, missing the cycle length in the exit part with a broad region of endocardial breakthrough **(Video 1)**.

Following this ablation attempt, VT was terminated and was noninducible with programmed extrastimulation with four extrastimuli down to refractoriness in the baseline state. An induction attempt was repeated with 20 μg/min of isoprenaline and with burst pacing down to 220 ms in the baseline state. Isoprenaline was initially commenced at 10 μg/min with a 2-μg bolus, with the programmed extrastimulation and RV burst-pacing protocol repeated after incrementing the isoprenaline dose by 10 μg/min up to the highest tolerated dose (20 μg/min). No recurrence of VT for at least two years of follow-up has been observed. This case provides a unique example of a three-dimensional VT circuit containing an epicardial, intramural component and an endocardial breakout.

## Discussion

Reentry is the predominant mechanism that sustains VT in the setting of ischemic cardiomyopathy and has provided the basic paradigm for understanding and guiding curative ablation. In general, reentrant circuits in ischemic cardiomyopathy have largely been localized to the endocardium or subendocardium. One of the explanations for this is the anatomy of the coronary artery. Specifically, blood in the coronary artery flows from the epicardium to endocardium; therefore, the endocardium is the most downstream tissue relative to the coronary blood flow, which is thought to be vulnerable to infarction. However, for a certain proportion of reentrant VT in patients with ischemic cardiomyopathy, mid- or subepicardial components could be a critical portion of the VT isthmus. Tschabrunn et al. reported that the VT substrate of animal infarct models may involve multiple myocardial layers.^3^ According to their study, scar first involves the subendocardium at the initial stage and then extends to the mid- or subepicardium later in the investigated animal infarct models. Recent studies have observed critical electrophysiologic epicardial substrate in 14%^4^ of cases, while data from cardiac magnetic resonance imaging (MRI) support the existence of an epicardial arrhythmogenic substrate in 64% of patients with transmural infarction, raising the profile of an epicardial approach for the treatment of VT in ischemic cardiomyopathy.^5^ Ashikaga et al. evaluated the role of cardiac MRI in a swine model of infarct-related VT. Following infarction and MRI, one group of animals underwent epicardial mapping and another underwent endocardial mapping only. While only 19% VTs in the epicardial group showed epicardial reentry, the critical isthmus was located at the scar border zone where small amounts of viable myocardium were bound by scar or lay over the infarct.^6^

**Figure 7:**
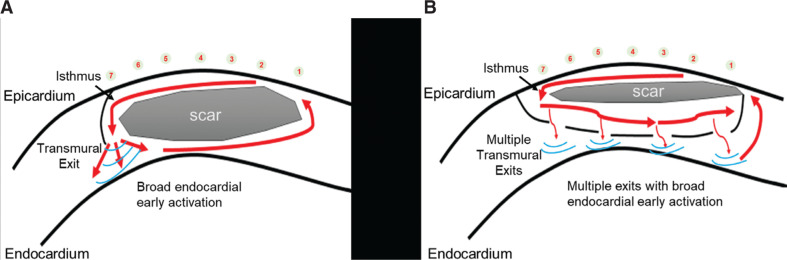
Potential mechanisms. **A:** Epicardial circuit with transmural exit. **B:** Epicardial circuit with multiple transmural exits.

In this case, we observed a macro-reentrant mechanism involving epicardial components and endocardial exit in a patient with ischemic cardiomyopathy. To our knowledge, there are scant reports in the literature of epicardial reentry with remote endocardial breakout. Broad regions of early endocardial activation invoke two potential mechanisms: the first involves reentry with a transmural exit that spreads radially from the exit toward the endocardium, enabling breakout to subtend a broad region of early endocardial activation **(Figure 7A)**, while, second, the VT substrate may involve multiple channels that exit over a broad region of endocardium and are sequentially activated as reentry ensues **(Figure 7B)**. In both instances, endocardial ablation of exit sites may subtly change the VT exit but fail to terminate VT.

Classical principles of entrainment definitively proved proximity to the circuit and ultimately guided the definition of the epicardial critical isthmus site and their successful ablation.^1,7^ This observation serves to highlight the futility of endocardial ablation at exit sites without a clear definition of the reentrant circuit.

Some investigators have advocated for a first-line endocardial–epicardial approach to VT ablation when noninvasive imaging reveals transmural scar.^8^ In contrast, others have suggested that an initial endocardial approach with the ablation of accessible abnormal potentials may obviate the need for epicardial ablation.^9^ A meta-analysis has reported the benefit of a combined endocardial–epicardial approach relative to an endocardial-only approach in ischemic cardiomyopathy.^10^ Though a combined approach is associated with fewer readmissions for VT or repeat ablations, there is an increase in complications as compared with following endocardial-only ablation.^11,12^ In the present case, detailed endocardial mapping of both ventricles and the aortic cusps failed to identify signals that were as compelling as the mid-diastolic potentials identified at the epicardial surface. This patient would have benefited from an earlier epicardial approach.

## Conclusions

This case highlights the three-dimensional nature of reentrant circuits in VT and the potential for epicardial reentry with endocardial breakout as a mechanism of postinfarction VT. It also emphasizes the need to consider an epicardial approach to ischemic VT ablation when endocardial ablation proves ineffective. Despite relatively early activation at endocardial sites, ablation will fail where ablation sites do not interrupt the reentrant circuit central to arrhythmogenesis. Where VT is hemodynamically tolerated, entrainment together with substrate mapping remain important for delineating critical sites amenable to ablation.

## Figures and Tables

**Video 1:** Epicardial reentry with endocardial (intramural) breakout.
